# A phase II study of daily encorafenib in combination with biweekly cetuximab in patients with BRAF V600E mutated metastatic colorectal cancer: the NEW BEACON study

**DOI:** 10.1186/s12885-022-10420-x

**Published:** 2022-12-16

**Authors:** Martina Eriksen, Per Pfeiffer, Kristoffer Staal Rohrberg, Christina Westmose Yde, Lone Nørgård Petersen, Laurids Østergaard Poulsen, Camilla Qvortrup

**Affiliations:** 1 Department of Oncology, Copenhagen University Hospital (Rigshospitalet), Copenhagen, Denmark; 2 Faculty of Health and Medical Sciences, University of Copenhagen, Copenhagen, Denmark; 3 Department of Oncology, Odense University Hospital, Odense, Denmark; 4 Department of Genomic Medicine, Copenhagen University Hospital (Rigshospitalet), Copenhagen, Denmark; 5 Department of Oncology, Aalborg University Hospital, Aalborg, Denmark

**Keywords:** Colorectal cancer, BRAF V600E mutation, BRAF targeted therapy, Biweekly cetuximab, Resistance, Genomic profiling

## Abstract

**Background:**

Patients with BRAF V600E mutated metastatic colorectal cancer (mCRC) have a poor prognosis. The introduction of BRAF targeted therapy with encorafenib and weekly administered cetuximab have shown improved survival with a median progression free survival (PFS) of 4.3 months. However, a regimen with cetuximab given every second week may have comparable efficacy and is more convenient for patients. While BRAF targeted therapy is a new standard therapy in pre-treated patients with BRAF V600E mutated mCRC, resistance invariably occurs and is an emerging challenge. The aim of this study is to investigate the efficacy and tolerability of cetuximab given every second week in combination with daily encorafenib and to explore the correlation between markers of resistance and outcome.

**Methods:**

The study is an open label, single arm, phase II study, investigating the efficacy and tolerability of cetuximab given every second week in combination with encorafenib in patients with BRAF V600E mutated mCRC. Furthermore, we will be investigating mechanisms of response and resistance against BRAF targeted therapy though comprehensive genomic profiling on tumor tissue and blood for circulating tumor DNA analysis. A total of 53 patients (19 + 34 in two steps) will be included according to Simon’s optimal two stage design. The primary end point of the study is 2 months PFS rate.

**Discussion:**

By combining BRAF inhibitor with cetuximab given every second week we can halve the number of visits in the hospital compared to the currently approved regimen with weekly cetuximab. This seems particularly relevant in a group of patients with a median overall survival of 9.3 months. Resistance after initial response to targeted therapy can be either adaptive (e.g., epigenetic, or transcriptomic alterations) or acquired (selective genetic alterations - e.g., activating de novo mutations) resistance. It is of great importance to untangle these complex mechanisms of resistance in patients with BRAF V600E mutated mCRC to improve treatment strategies in the future potentially even further.

**Trial registration:**

EU Clinical Trial Register, Eudract no. 2020-003283-10. Registered on 11 November 2020.

## Background

Colorectal cancer (CRC) is among the most prevalent cancer diseases worldwide accounting annually for more than 1.900.000 new cases, and around 940.000 deaths [1]. Despite optimized surgical procedures and adjuvant combination chemotherapy, many patients still experience disease recurrence, most often with a fatal course. CRC is a heterogeneous disease based on its genetics, but for many years treatment of metastatic CRC (mCRC) has been limited to chemotherapy alone. However, in recent decades the treatment strategies have evolved because of the progress in our understanding of cancer as a disease of the genome and advances in molecular diagnostics. In mCRC, the significance of being able to detect *RAS* mutational status is one example that have led to improved results with the introduction of monoclonal antibodies (such as cetuximab and panitumumab) [2–4] – in 2013 the therapeutic indication was restricted by the European Medicines Agency (EMA) to patients with *RAS* wild-type mCRC tumors only [5]. Later it became possible to target tumors with deficient mismatch repair (dMMR) which often (in sporadic CRC cases) is caused by methylation of the *MLH1* gene promotor [6–8]. Based on the KEYNOTE-177 trial [9] programmed death 1 (PD-1) blockade with pembrolizumab is now standard of care to patients with dMMR mCRC.

Targeting BRAF in CRC has been under investigation [10–12] and this is highly clinically relevant since patients with mutated BRAF V600E mCRC have a very poor prognosis [13].

*BRAF* encodes the BRAF protein and a missense mutation of the gene – the single nucleotide substitution (T > A at codon 1799), resulting in the protein variant V600E – accounts for the vast majority of the *BRAF* mutations*.* The BRAF V600E mutation results in a constitutively activation of the mitogen-activated protein kinase (MAPK) pathway, which drives cellular proliferation and survival [14]. The incidence of BRAF V600E mutation in CRC is described to be around 10% in patients in clinical trials [15], but as high as 20% in unselected Nordic populations [16, 17].

Response to treatment with BRAF inhibitors (BRAFi) is extremely dependent on the origin of the *BRAF* mutated tumor. BRAF V600E mutated melanoma respond to BRAFi monotherapy with response rates (RR) of more than 50% [18] and in combination with MEK inhibitor (MEKi) survival is further prolonged [19–21]. In mCRC, BRAFi has very limited efficacy as monotherapy (RR around 5% [22]) and in combination with MEKi, whereas, targeting BRAF in combination with epidermal growth factor receptor (EGFR) has shown promising antitumor activity and survival benefits in the BEACON trial [12], where pre-treated patients with BRAF V600E mutated mCRC were randomized to receive treatment with either encorafenib with weekly cetuximab (doublet BRAF targeted therapy) or encorafenib with weekly cetuximab and binimetinib (triplet BRAF targeted therapy) or investigator’s choice of chemotherapy-based treatment.

Triplet therapy increased RR to approximately 26% and median progression free survival (PFS) to 4.3 months – and updated analysis found that doublet and triplet therapy resulted in similar overall efficacy across endpoints including PFS and overall survival (OS) [23]. These results led the United States Food and Drug Administration (FDA) to approve the doublet regimen: Cetuximab administered weekly in combination with encorafenib, on April 8th, 2020, to pre-treated patients with BRAF V600E mutated mCRC. The regimen with weekly cetuximab was approved by EMA in June 2020 and is considered a new standard therapy to patients with pre-treated BRAF V600E mutated mCRC.

A regimen with weekly intravenous therapy entails many visits in the hospital, however a regimen where cetuximab is given once every second week at a double dose, seems to have efficacy and tolerability comparable to weekly therapy, and is much more convenient for patients [24–31].

While BRAF targeted therapy in mCRC is a new standard of care, with a significant antitumor activity, rapid resistance against the therapy is an emerging challenge. It is of great importance to understand these mechanisms of resistance to ensure further development within the field of personalized therapy for patients with BRAF mutated CRC.

### Rationale of the study

The purpose of this study is to evaluate the efficacy and tolerability of biweekly cetuximab combined with standard dose encorafenib in patients with pre-treated BRAF V600E mutated mCRC. Furthermore, we wish to obtain a better understanding of mechanisms of response and resistance to BRAF targeted therapy in patients with mCRC.

## Design and methods

### Study design

The study is an open label, single arm, multicenter, phase II study.

### Eligible criterias

All patients must provide written informed content before inclusion in the study.

Eligible patients must be above the age of 18 years, WHO performance status of 0-1, and have histologically verified BRAF V600E mutated CRC, adenocarcinoma, proficient mismatch repair (pMMR), non-resectable and/or metastatic disease, and they must have received prior systemic treatment for CRC.

Measurable and/or evaluable non-measurable disease, and tumor lesion accessible for biopsy, is mandatory. Furthermore, included patients must have adequate haematological, cardiac and organ function. Finally, prior treatment with any EGFR-, RAF- or MEK-inhibitor is prohibited, and the patient is not allowed to have any known activating *RAS* mutation present at baseline.

### Treatment

The patients will be treated in cycles of 28 days.

Encorafenib will be administered once daily, orally, as a fixed dose, 300 mg once daily day 1-28.

Cetuximab will be administered as intravenous infusion on study site according to institutional standards (which includes premedication prior to each infusion), in dosage 500 mg/m^2^ every second week on day 1 and day 15 of each cycle.

Treatment will continue until progressive disease (PD), unacceptable adverse effects or patients wish of ending treatment.

Before starting study treatment, a baseline computed tomography (CT) scan will be performed, and new CT scans will be performed after every second cycle to evaluate response to treatment.

Response to treatment will be evaluated – when possible – according to the Response Evaluation Criteria in Solid Tumors (RECIST) version 1.1.

### End points

The primary endpoint of the study is 2 months PFS rate. Secondary endpoints include OS, PFS, RR according to RECIST version 1.1 – in patients with measurable disease – and toxicity.

Exploratory endpoints are correlation between tumor markers, markers of resistance and markers of tumor evolution and outcome.

### Statistics

The sample size is based on Simon’s optimal two stages design [32]. This design ensures early study termination if there is insufficient effect.

Patients with BRAF V600E mutated mCRC are at high risk of immediate progression with standard chemotherapy. In the BEACON study, the 2 months PFS rate was around 84%. A 2 months PFS rate less than 60% is not considered clinically acceptable. Assuming a significance level at 0.05 (α = 0.05) and a power at 90% (β = 0.10) it can be calculated that 19 patients should be included in the first part of the study. The enrollment will continue until 19 patients have received 2 months of therapy. If 12 or less out of the first 19 patients continue therapy beyond 2 months without PD, we will reject our hypothesis and close the study after the first stage of accrual.

If 13 or more patients continue therapy for at least 2 months without PD, an additional 34 patients will be accrued in the second stage. If at least 38 out of 53 patients continue therapy beyond 2 months without PD we will conclude that the treatment is effective enough to continue with future studies.

PFS and OS will be estimated using the Kaplan-Meier method. PFS will be calculated from time of trial inclusion till progression either by RECIST, clinically or death. OS will be calculated from time of trial inclusion to death.

### Biopsies and circulating tumor DNA (ctDNA) analysis

Fresh tumor biopsies, primarily from metastatic lesions, are obtained from patients at baseline, after two cycles of treatment (on-treatment), and upon progression of disease. Biopsies are either core needle biopsies (18-gauge needle) or surgical resections samples. Three samples are taken from the same lesion each time – one sample is formalin-fixed and paraffin-embedded (FFPE) for histopathological verification of the tissue and two samples, stored in RNAlater, are for comprehensive genomic analyses. A blood sample is collected at baseline and used for subtraction of germline variants to identify tumor specific mutations only.

Blood samples (collected in STRECK-tubes) for analysis of ctDNA are taken prior to each treatment cycle.

### Genomic profiling

DNA and RNA from each biopsy is extracted and purified to perform whole genome sequencing (WGS), RNA sequencing (RNAseq), and single nucleotide polymorphism (SNP) arrays. WGS is performed from tumor and germline DNA using Illumina polymerase chain reaction (PCR) free DNA prep, and RNAseq is performed from tumor RNA using Illumina Stranded Total RNA Prep. SNP array is performed with CytoScan HD/OncoScan array. The DNA and RNA libraries are sequenced on NovaSeq6000 (Illumina) as 2 × 150 bp paired-end sequencing. The raw data is mapped to the hg38/GRCh38 human reference genome using BWA-MEM v0.7.12 software and bioinformatics is done following Genome Analysis Toolkit (GATK) best practice.

Somatic mutations are identified by subtracting the germline variants from the tumor variants. RNAseq is performed to identify gene fusions and to evaluate expression levels of selected, predefined targets. SNP array analysis is performed to identify copy number alterations.

### Ethics

The study is being conducted in accordance with the protocol, the ethical principles of the Helsinki Declaration and in accordance with good clinical practice (GCP) and regulations of Research Ethics Committee.

This study was approved by the Ethics Committee (1-10-72-239-20) and by the Danish Medicines Agency (Eudract 2020-003283-10).

## Genomic research

Prior to enrolment into the study, the patient will receive oral and written information regarding genomic profiling and the risk of incidental findings.

The patient will have the right not to know of incidental findings if preferred.

All genomic reports will be discussed on the National Molecular Tumor Board with participation of clinical oncologists, clinical geneticists, pathologists, and molecular biologists. Any incidental findings will be handled based on recommendations made by the National Molecular Tumor Board.

## Discussion

Patients with BRAF V600E mCRC treated with BRAF targeted therapy have a median OS of around 9 months [12]. With limited lifetime expectancy it seems very important to try to minimize time spend in the hospital for these patients. With a BRAF targeted regimen with cetuximab given every second week we can halve the number of visits to the hospital compared to the currently approved regimen with weekly cetuximab. We therefore find it highly relevant to investigate the efficacy and tolerability of cetuximab given every second week, together with encorafenib, in the current study.

Resistance against BRAF targeted therapy is a clinically significant challenge. Despite initial response to BRAFi resistance invariably occurs. Mechanisms of resistance has been untangled to some degree in melanoma [33, 34]. The reason why some primary tumors are widely unresponsive to BRAFi monotherapy, even though BRAF V600E mutation is present, is largely unknown [35].

In BRAF V600E mutated mCRC some of the resistance to BRAFi monotherapy may be explained by EGFR-mediated reactivation of MAPK pathway [10, 36] which is managed by combining BRAFi with an anti-EGFR antibody (e.g. cetuximab). However, resistance against BRAF targeted therapy in patients with BRAF V600E mutated mCRC remains a serious problem, as the PFS is just around 4 months [12].

Resistance to BRAF targeted therapy – *after* initial response – can be either due to adaptive resistance or acquired resistance [37]. Adaptive resistance can be de novo adaption of cellular epigenetic and transcriptomic changes and has a rapid time frame, where acquired resistance arises due to selective genetic alterations coming from the therapy and/or the acquisition of therapy-induced de novo alterations [38]. In most situations the resistance against BRAF targeted therapy happens through reactivation of the MAPK pathway: examples are activating mutations (e.g., in *NRAS*), *BRAF* amplifications or CRAF overexpression [38]. Mechanisms of acquired resistance can also be found outside the MAPK pathway – one example is signaling through the PI3K-AKT pathway [39].

The incomplete and transient nature of response to targeted therapy is described to be due to the residual state of disease in tumors that are not eliminated by therapy [40]. From the residual disease subsequent tumor progression and tumor evolution can occur and resistance may arise.

The understanding of resistance against BRAF targeted therapy is complex and further research is warranted. The comprehensive genomic profiling performed throughout the treatment in our study, including the ctDNA analyses, has the potential to gain valuable insights regarding tumor evolution and mechanisms of resistance to BRAF targeted therapy in BRAF V600E mutated mCRC.

## Status of the study

The study was initiated at Rigshospitalet, Copenhagen, Denmark in February 2021. Other currently active sites are Odense University Hospital, Denmark and Aalborg University Hospital, Denmark.

To date 13 patients have been included in the study.

## Data Availability

The datasets generated and/or analyzed during the current study are not publicly available due to that this is an ongoing trial.

## Abbreviations

BRAFi: BRAF Inhibitor
CRC: Colorectal Cancer
CT: Computed Tomography
ctDNA: Circulating Tumor DNA
dMMR: Deficient Mismatch Repair
EGFR: Epidermal Growth Factor Receptor
EMA: European Medicines Agency
FDA: Food and Drug Administration
FFPE: Formalin-Fixed and Paraffin-Embedded
GATK: Genome Analysis ToolKit
GCP: Good Clinical Practice
MAPK: Mitogen-Activated Protein Kinase
mCRC: Metastatic Colorectal Cancer
MEKi: MEK Inhibitor
OS: Overall Survival
PCR: Polymerase Chain Reaction
PD: Progressive Disease
PD-1: Programmed Cell Death 1
PFS: Progression Free Survival
pMMR: Proficient Mismatch Repair
RECIST: Response Evaluation Criteria in Solid Tumors
RNAseq: RNA sequencing
RR: Response Rate
SNP: Single Nucleotide Polymorphism
WGS: Whole Exome Sequencing

## References

[1] Woods P. Colorectal cancer statistics [Internet]. WCRF International. [cited 2022 Jul 4]. Available from: https://www.wcrf.org/cancer-trends/colorectal-cancer-statistics/

[2] Van Cutsem E, Ch K, Schlichting M, Zubel A, Celik I, Rougier P (2011). Cetuximab plus irinotecan, fluorouracil, and Leucovorin as first-line treatment for metastatic colorectal Cancer: updated analysis of overall survival according to tumor KRAS and BRAF mutation status. J Clin Oncol.

[3] Douillard JY, Oliner KS, Siena S, Tabernero J, Burkes R, Barugel M (2013). Panitumumab–FOLFOX4 treatment and RAS mutations in colorectal Cancer. N Engl J Med.

[4] Peeters M, Price TJ, Cervantes A, Sobrero AF, Ducreux M, Hotko Y (2010). Randomized phase III study of Panitumumab with fluorouracil, Leucovorin, and irinotecan (FOLFIRI) compared with FOLFIRI alone as second-line treatment in patients with metastatic colorectal Cancer. J Clin Oncol.

[5] Dufraing K, Keppens C, Tack V, Siebers AG, Kafatos G, Dube S, et al. Evolution of RAS testing over time: factors influencing mutation rates in metastatic colorectal cancer patients. Colorectal Cancer. 2020;9(1):CRC14. 10.2217/crc-2019-0013.

[6] Koopman M, Kortman GAM, Mekenkamp L, Ligtenberg MJL, Hoogerbrugge N, Antonini NF (2009). Deficient mismatch repair system in patients with sporadic advanced colorectal cancer. Br J Cancer.

[7] Arnold CN, Goel A, Compton C, Marcus V, Niedzwiecki D, Waserman L (2003). Evaluation of microsatellite instability, hMLH1 expression and hMLH1 promoter hypermethylation in defining the phenotype of colorectal cancer: a study from CALGB 9865. Gastroenterology (New York, NY 1943).

[8] Goel A, Boland CR (2012). Epigenetics of colorectal Cancer. Gastroenterology (New York, NY 1943).

[9] André T, Shiu KK, Kim TW, Jensen BV, Jensen LH, Punt C (2020). Pembrolizumab in microsatellite-instability–high advanced colorectal Cancer. N Engl J Med.

[10] Prahallad A, Sun C, Huang S, Di Nicolantonio F, Salazar R, Zecchin D (2012). Unresponsiveness of colon cancer to BRAF(V600E) inhibition through feedback activation of EGFR. Nature (London).

[11] Van Cutsem E, Huijberts S, Grothey A, Yaeger R, Cuyle PJ, Elez E (2019). Binimetinib, Encorafenib, and Cetuximab triplet therapy for patients with BRAF V600E-mutant metastatic colorectal Cancer: safety Lead-in results from the phase III BEACON colorectal Cancer study. J Clin Oncol.

[12] Kopetz S, Grothey A, Yaeger R, Van Cutsem E, Desai J, Yoshino T (2019). Encorafenib, Binimetinib, and Cetuximab in BRAF V600E–mutated colorectal Cancer. N Engl J Med.

[13] Tran B, Kopetz S, Tie J, Gibbs P, Jiang ZQ, Lieu CH (2011). Impact of BRAF mutation and microsatellite instability on the pattern of metastatic spread and prognosis in metastatic colorectal cancer. Cancer..

[14] Davies H, Bignell GR, Cox C, Stephens P, Edkins S, Clegg S (2002). Mutations of the BRAF gene in human cancer. Nature..

[15] Caputo F, Santini C, Bardasi C, Cerma K, Casadei-Gardini A, Spallanzani A (2019). BRAF-mutated colorectal Cancer: clinical and molecular insights. Int J Mol Sci.

[16] Sorbye H, Dragomir A, Sundström M, Pfeiffer P, Thunberg U, Bergfors M (2015). High BRAF mutation frequency and marked survival differences in subgroups according to KRAS/BRAF mutation status and tumor tissue availability in a prospective population-based metastatic colorectal Cancer cohort. Plos one.

[17] Winther SB, Liposits G, Skuladottir H, Hofsli E, Shah CH, Poulsen LØ (2019). Reduced-dose combination chemotherapy (S-1 plus oxaliplatin) versus full-dose monotherapy (S-1) in older vulnerable patients with metastatic colorectal cancer (NORDIC9): a randomised, open-label phase 2 trial. Lancet Gastroenterol Hepatol.

[18] McArthur GA, Chapman PB, Robert C, Larkin J, Haanen JB, Dummer R (2014). Safety and efficacy of vemurafenib in BRAF(V600E) and BRAF(V600K) mutation-positive melanoma (BRIM-3): extended follow-up of a phase 3, randomised, open-label study. Lancet Oncol.

[19] Flaherty KT, Infante JR, Daud A, Gonzalez R, Kefford RF, Sosman J (2012). Combined BRAF and MEK inhibition in melanoma with BRAF V600 mutations. N Engl J Med.

[20] Long GV, Stroyakovskiy D, Gogas H, Levchenko E, de Braud F, Larkin J (2015). Dabrafenib and trametinib versus dabrafenib and placebo for Val600 BRAF-mutant melanoma: a multicentre, double-blind, phase 3 randomised controlled trial. Lancet..

[21] Ascierto PA, McArthur GA, Dréno B, Atkinson V, Liszkay G, Di Giacomo AM (2016). Cobimetinib combined with vemurafenib in advanced BRAF(V600)-mutant melanoma (coBRIM): updated efficacy results from a randomised, double-blind, phase 3 trial. Lancet Oncol.

[22] Kopetz S, Desai J, Chan E, Hecht JR, O’Dwyer PJ, Maru D (2015). Phase II pilot study of Vemurafenib in patients with metastatic BRAF-mutated colorectal Cancer. J Clin Oncol.

[23] Tabernero J, Grothey A, Van Cutsem E, Yaeger R, Wasan H, Yoshino T (2021). Encorafenib plus Cetuximab as a new standard of Care for Previously Treated BRAF V600E-mutant metastatic colorectal Cancer: updated survival results and subgroup analyses from the BEACON study. J Clin Oncol.

[24] Pfeiffer P, Nielsen D, Bjerregaard J, Qvortrup C, Yilmaz M, Jensen B (2008). Biweekly cetuximab and irinotecan as third-line therapy in patients with advanced colorectal cancer after failure to irinotecan, oxaliplatin and 5-fluorouracil. Ann Oncol.

[25] Tabernero J, Pfeiffer P, Cervantes A (2008). Administration of Cetuximab every 2 weeks in the treatment of metastatic colorectal Cancer: an effective, more convenient alternative to weekly administration?. Oncologist.

[26] Brodowicz T, Ciuleanu TE, Radosavljevic D, Shacham-Shmueli E, Vrbanec D, Plate S (2013). FOLFOX4 plus cetuximab administered weekly or every second week in the first-line treatment of patients with KRAS wild-type metastatic colorectal cancer: a randomized phase II CECOG study. Ann Oncol.

[27] Pietrantonio F, Di Bartolomeo M, Cotsoglou C, Mennitto A, Berenato R, Morano F (2016). Perioperative triplet chemotherapy and cetuximab in patients with RAS wild-type high recurrence risk or borderline resectable colorectal cancer liver metastases. Clin Colorectal Cancer.

[28] Spindler KLG, Pallisgaard N, Appelt AL, Andersen RF, Schou JV, Nielsen D (2015). Clinical utility of KRAS status in circulating plasma DNA compared to archival tumour tissue from patients with metastatic colorectal cancer treated with anti-epidermal growth factor receptor therapy. Eur J Cancer.

[29] Pfeiffer P, Sorbye H, Qvortrup C, Karlberg M, Kersten C, Vistisen K (2015). Maintenance therapy with Cetuximab every second week in the first-line treatment of metastatic colorectal Cancer: the NORDIC-7.5 study by the Nordic colorectal Cancer biomodulation group. Clin Colorectal Cancer.

[30] Rossini D, Santini D, Cremolini C, Salvatore L, Lonardi S, Dell’Aquila E (2017). Rechallenge with cetuximab + irinotecan in 3rd-line in RAS and BRAF wild-type metastatic colorectal cancer (mCRC) patients with acquired resistance to 1st-line cetuximab+irinotecan: the phase II CRICKET study by GONO. Ann Oncol.

[31] Cremolini C, Antoniotti C, Lonardi S, Aprile G, Bergamo F, Masi G (2018). Activity and safety of Cetuximab plus modified FOLFOXIRI followed by maintenance with Cetuximab or bevacizumab for RAS and BRAF wild-type metastatic colorectal Cancer: a randomized phase 2 clinical trial. JAMA Oncol.

[32] Simon R (1989). Optimal two-stage designs for phase II clinical trials. Control Clin Trials.

[33] Moriceau G, Hugo W, Hong A, Shi H, Kong X, Yu CC (2015). Tunable-combinatorial mechanisms of acquired resistance limit the efficacy of BRAF/MEK Cotargeting but result in melanoma drug addiction. Cancer Cell.

[34] Villanueva MT (2015). Therapeutic resistance: paradox breaking. Nat Rev Cancer.

[35] Baik CS, Myall NJ, Wakelee HA (2017). Targeting BRAF-mutant non-small cell lung Cancer: from molecular profiling to rationally designed therapy. Oncologist.

[36] Corcoran RB, Ebi H, Turke AB, Coffee EM, Nishino M, Cogdill AP (2012). EGFR-mediated reactivation of MAPK signaling contributes to insensitivity of BRAF -mutant colorectal cancers to RAF inhibition with Vemurafenib. Cancer Discovery.

[37] Holderfield M, Deuker MM, McCormick F, McMahon M (2014). Targeting RAF kinases for cancer therapy: BRAF-mutated melanoma and beyond. Nat Rev Cancer.

[38] Zaman A, Wu W, Bivona TG (2019). Targeting oncogenic BRAF: past, present, and future. Cancers..

[39] Johnson DB, Menzies AM, Zimmer L, Eroglu Z, Ye F, Zhao S (2015). Acquired BRAF inhibitor resistance: a multicenter meta-analysis of the spectrum and frequencies, clinical behaviour, and phenotypic associations of resistance mechanisms. Eur J Cancer.

[40] Bivona TG, Doebele RC (2016). A framework for understanding and targeting residual disease in oncogene-driven solid cancers. Nat Med.

